# A fully automated deep learning approach for coronary artery segmentation and comprehensive characterization

**DOI:** 10.1063/5.0181281

**Published:** 2024-01-23

**Authors:** Guido Nannini, Simone Saitta, Andrea Baggiano, Riccardo Maragna, Saima Mushtaq, Gianluca Pontone, Alberto Redaelli

**Affiliations:** 1 Department of Electronics Information and Bioengineering, Politecnico di Milano, Milan, Italy; 2 Department of Perioperative Cardiology and Cardiovascular Imaging D, Centro Cardiologico Monzino IRCCS, Italy; 3 Department of Clinical Sciences and Community Health. University of Milan, Milan, Italy; 4 Department of Biomedical, Surgical and Dental Sciences, University of Milan, Milan, Italy

## Abstract

Coronary computed tomography angiography (CCTA) allows detailed assessment of early markers associated with coronary artery disease (CAD), such as coronary artery calcium (CAC) and tortuosity (CorT). However, their analysis can be time-demanding and biased. We present a fully automated pipeline that performs (i) coronary artery segmentation and (ii) CAC and CorT objective analysis. Our method exploits supervised learning for the segmentation of the lumen, and then, CAC and CorT are automatically quantified. 281 manually annotated CCTA images were used to train a two-stage U-Net-based architecture. The first stage employed a 2.5D U-Net trained on axial, coronal, and sagittal slices for preliminary segmentation, while the second stage utilized a multichannel 3D U-Net for refinement. Then, a geometric post-processing was implemented: vessel centerlines were extracted, and tortuosity score was quantified as the count of branches with three or more bends with change in direction forming an angle >45°. CAC scoring relied on image attenuation. CAC was detected by setting a patient specific threshold, then a region growing algorithm was applied for refinement. The application of the complete pipeline required <5 min per patient. The model trained for coronary segmentation yielded a Dice score of 0.896 and a mean surface distance of 1.027 mm compared to the reference ground truth. Tracts that presented stenosis were correctly segmented. The vessel tortuosity significantly increased locally, moving from proximal, to distal regions (p < 0.001). Calcium volume score exhibited an opposite trend (p < 0.001), with larger plaques in the proximal regions. Volume score was lower in patients with a higher tortuosity score (p < 0.001). Our results suggest a linked negative correlation between tortuosity and calcific plaque formation. We implemented a fast and objective tool, suitable for population studies, that can help clinician in the quantification of CAC and various coronary morphological parameters, which is helpful for CAD risk assessment.

## I. INTRODUCTION

Coronary artery disease (CAD) is the most frequent cardiovascular disease and the leading cause of death worldwide.^1^ CAD results from the buildup of fibro-lipidic and calcific plaques in the coronary arteries, leading to stenosis and reduced blood flow, potentially causing myocardial ischemia.^2^

Coronary computed tomography angiography (CCTA) has emerged as the primary imaging technique for the assessment of coronary arteries, providing high resolution images that allow a thorough analysis of the vessel lumen.^3^ With respect to other imaging techniques, such as invasive coronary angiography and intravascular ultrasound, CCTA enables a less invasive assessment of the whole coronary circulation, with a single volumetric acquisition. The analysis of CCTA is the basis for the identification of early markers and the understanding of factors associated with CAD progression, to achieve effective risk stratification and preventive intervention.

Coronary artery calcium (CAC) is an indicator of atherosclerosis, and it has been proven in different studies^4–6^ that high levels of CAC are associated with an increased risk of adverse cardiovascular events. In clinical practice, CAC is quantified from a specific CT acquisition, namely, the calcium scoring CT (CSCT), by identifying groups of connected voxels with attenuation above 130 Hounsfield units (HU).^7^ Pavitt *et al.*^8^ and Mylonas *et al.*^9^ were the first to show excellent agreement between CAC scoring obtained from CSCT and CCTA, using patient-specific attenuation thresholds to detect calcium. Indeed, in CCTA, calcium attenuation takes higher values, and defining a unique threshold is not possible due to variations in lumen attenuation caused by the acquisition protocol or the contrast agent. Thus, the identification of calcific lesions still relies on an expert manual analysis for the setting of the correct threshold. In clinical practice, the standard scoring metrics of CAC are the volume score (VS) and the percentage of plaque volume (PPV) with respect to the volume of the coronary artery.^10,11^ CAC scoring is generally performed by semi-automatic tools.

Beyond CAC itself, the morphological features of coronary arteries also play a critical role in assessing the risk associated with CAD progression. Han *et al.*^6^ studied the association between the vessel geometric features in the regions where CAC forms and future adverse cardiovascular events, showing that, other than VS and PPV, adverse geometric characteristics such as short distance from the coronary ostium, proximity to bifurcations, and tortuosity of the vessel are significantly more exhibited by subjects that develop future adverse conditions. In particular, coronary tortuosity (CorT) is frequently observed in CCTA analysis, yet its etiology and clinical relevance are still unclear.^12–15^ CorT is quantified through the tortuosity score (TS), which is the number of main coronary branches that present three or more bends, with each bend entailing a change in the vessel direction greater than 45°. A TS = 0 indicates no CorT, while TS ≥ 4 indicates severe CorT. CorT is manually measured directly on medical images, and thus, findings from different studies may be subject to operator bias. Furthermore, CorT is measured on coronary angiography images or curved planar reformatted CCTA, each of which is a 2D representation of the vessel.

A prerequisite for the quantification of any marker associated with CAD progression is an accurate segmentation of the coronary lumen. Manual segmentation is typically performed by experts; however, this process may require up to 1 h and produce incomplete segmentations of subtle tracts that may be overlooked.

Recently, deep learning techniques have emerged as a compelling alternative to conventional image segmentation algorithms demonstrating comparable accuracy while consistently offering significant time saving. Once trained, generally complete it in less than 1 min. Convolutional neural networks (CNNs) are currently the most widely adopted tool for the segmentation of volumetric images. As compared to other organs, coronary arteries are particularly challenging to segment automatically, mainly due to their highly variable complex anatomy that presents many branches and an irregular course. Several CNN architectures for the automatic segmentation of coronary arteries, mostly based on the U-Net model,^16^ have been proposed. The nnU-Net^17^ and a 2.5D U-Net net were the models that yielded the best results in terms of precision, recall, and Dice similarity coefficient (DSC).^18^ To circumvent the large heterogeneity of medical data, due to anatomical variability, different authors have proposed to use additional information in input, such as the centerline,^19^ the vesselness image,^20^ or a rough segmentation,^21^ that allows to encode the position of coronary arteries, to improve the performance of the models.

In the present work, we developed a fully automated pipeline for the segmentation and characterization of coronary arteries in terms of CAC and CorT, which fed on CCTA images outputs the reconstructed vessel lumen and the analysis of CAC and CorT. The proposed method exploits, to the best of our knowledge, the largest dataset that has been used to train and test a CNN for the automatic reconstruction of coronary arteries, including patients with CAD resulting from various etiologies. Our pipeline is suitable for unbiased quantification and analysis of calcium and tortuosity features in populations studies, as it requires only medical images as input and no further interaction with the operator.

## II. RESULTS

In this section, we present the outcomes obtained through the implemented pipeline, covering the following aspects: (i) evaluating the CNN architecture's performance on the test set; (ii) extracting and validating features related to CorT and CAC; (iii) examining the relationship between CorT, CAC, and CAD risk factors; (iv) investigating the relation between CorT, CAC, and their location along the coronary centerline; and (v) exploring the interplay between CorT and CAC.

We emphasize that all statistical analyses pertaining to CorT and CAC relied solely on manual segmentations of the coronary arteries. Inference was run on the test stet cases to assess the CNN performance. The automated pipeline for CorT and CAC features extraction was then applied to the entire dataset, consisting of 281 manual annotations, to ensure statistical relevance of the subsequent analysis.

### A. Test set results

**FIG. 1.**
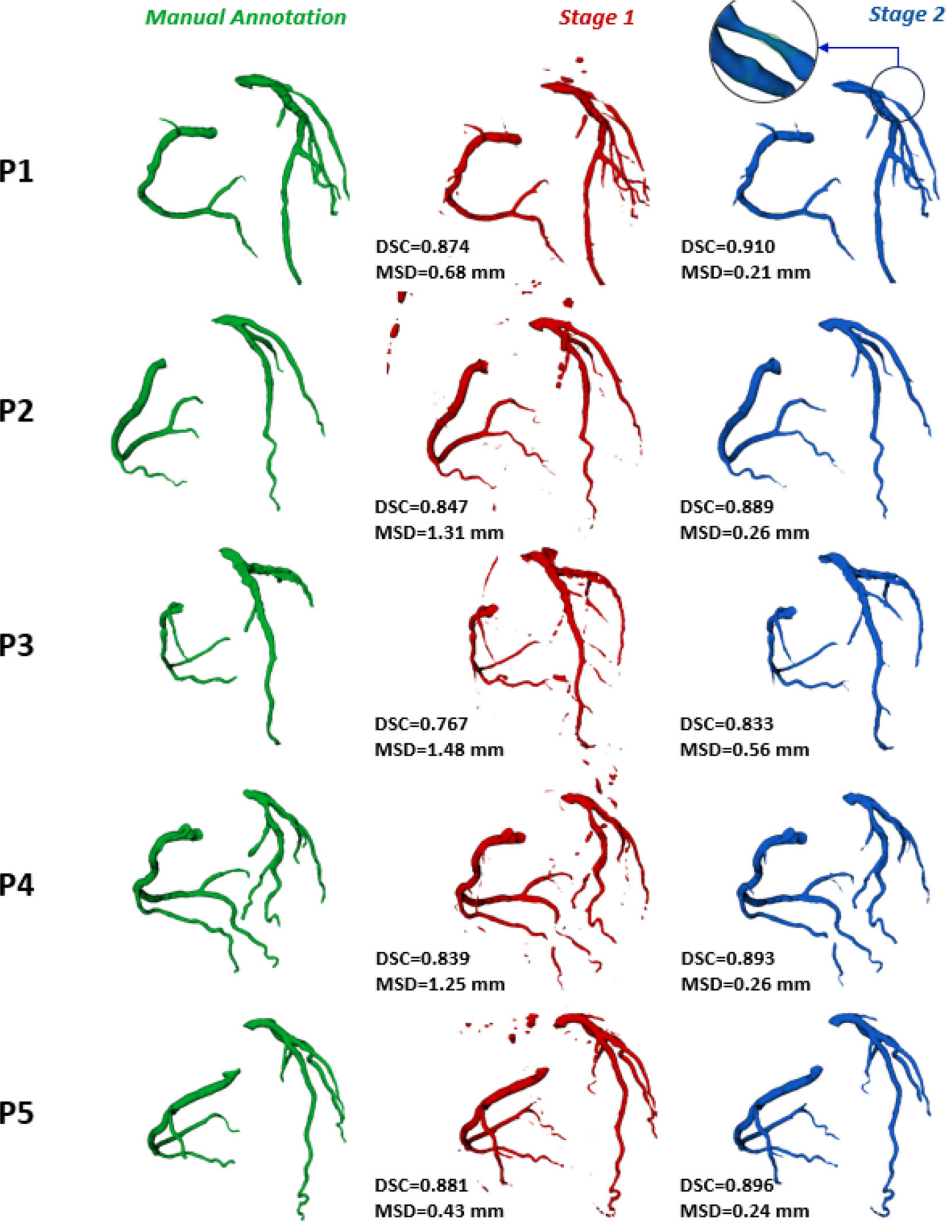
Comparison of results obtained with the stage 1 (S1) model and the stage 2 (S2) cascaded model on five test cases. From left to right: manual annotation and reference ground truth (in green), S1 prediction (in red), and S2 prediction. For the latter two, the Dice similarity coefficient (DSC) and mean surface distance (MSD) computed against the ground truth are reported in the lower left corner. For P1, a zoom-in on a stenotic region of the circumflex artery is provided, displaying the ground truth with transparency.

Once the cascaded-CNN was trained, inference was run on the 43 cases of the test set. Inference took ∼45 s to run on a 16GB GeForce RTX3060 GPU and ∼4 min to run on a 16 cores AMD Ryzen 3955WX CPU. A qualitative comparison vs the manual annotation of both S1 and S2 predictions is shown for five representative cases of the test set in Fig. 1: S2 segmentation resulted more accurate than S1 one for all the subject in the test set. It is notable that in patient P1, who presented a severe stenosis in the left circumflex artery, our model correctly segmented the stenotic region. To assess the impact of using a cascaded model, the output of the solely multi-view 2.5D model trained in S1 was compared to the output of the 3D cascaded model trained in the S2. Each performance metric was computed both for the first and second stage predictions, yielding an average DSC_S1_ equal to 0.791 [0.60; 0.88] and a DSC_S2_ equal to 0.895 [0.75; 0.92] (Δ = +13%); a MSD_S1_ of 1.801 [0.52; 3.33] mm and a MSD_S2_ of 0.470 [0.16; 0.86] mm (Δ = −73%). The average precision was 0.892 ± 0.053 and 0.935 ± 0.044 (Δ = +5%), while recall was 0.861 ± 0.041 and 0.891 ± 0.061 (Δ = +3.5%) for S1 and S2, respectively.

### B. Coronary tortuosity and calcium features extraction

**FIG. 2.**
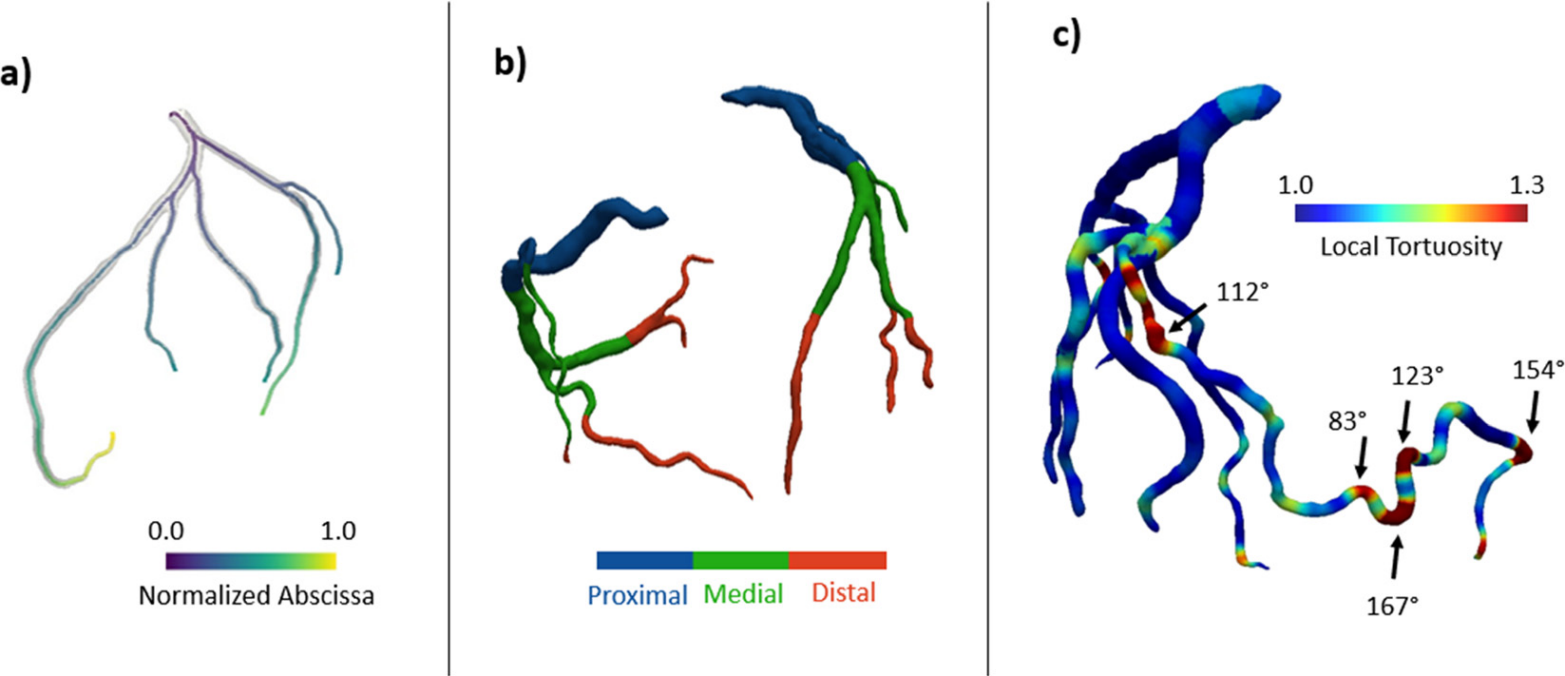
(a) Centerlines automatically extracted with the recursive approach described in Sec. II D. (b) Definition of proximal, medial, and distal segments of coronary arteries based on normalized geodesic distances. (c) Local tortuosity map showing in the dark red region with high tortuosity (i.e., sharp change in direction of the vessel), where the tortuosity angle is indicated.

Coronary centerlines, tortuosity features, and calcific plaque features were successfully extracted for each patient using the automated pipeline described in Sec. II D. The comprehensive analysis of CAC and CorT was achieved in ∼5 min per patient. Figure 2 illustrates the results obtained from the morphological analysis.

**FIG. 3.**
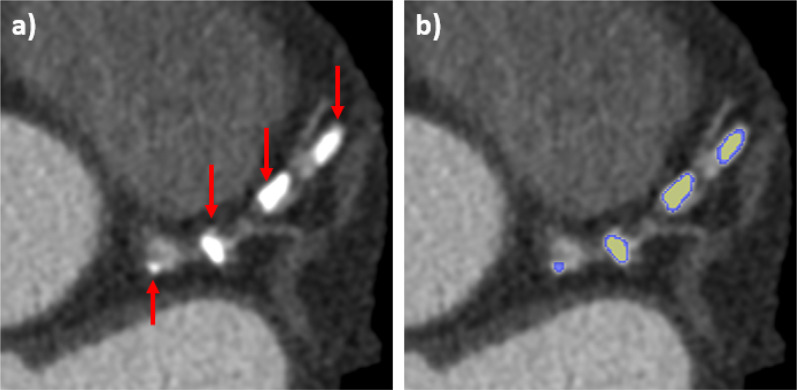
Example of coronary calcium detection: (a) CCTA of a patient presenting visible calcific plaques (indicated by arrows) in the left coronary artery and (b) segmentation of calcium before (yellow) and after the application of the region growing algorithm.

The output of the CAC detection algorithm is illustrated in Fig. 3 empathizing with different colors the under-segmentation produced by a simple thresholding algorithm with respect to our region growing-based approach. The Pearson correlation coefficient between CAC volume computed from CSCT and CCTA yielded a value of *r* = 0.96 (p < 0.001). CAC volume was generally lower in CCTA than in CSCT. From Bland–Altman analysis, the mean difference between scores (i.e., the bias) was −92.3 mm^3^ (95% limit of agreement −359.3 to 182.8).

### C. Relation between CAC and coronary artery disease risk factors

CAC features were compared between groups of patients based on the presence of CAD risk factors, in different anatomical segments of the coronary artery. For each anatomical portion, the average volume score and PPV are summarized in Table I. Calcium VS and PPV were significantly higher in old (p = 0.008, p = 0.021), hypertensive (p = 0.015, p = 0.049), and diabetic (p = 0.015, p = 0.019) patients. Old subjects exhibited a significant increase in calcium volume in the proximal and medial regions, while hypertensive and diabetic patients in the proximal region. Smoking patients presented a significant increment of calcium volume in the medial (p = 0.021) and distal regions (p = 0.027). The extent of the detected calcific lesions is reported in Table II. Old, smoking, and hypertensive patients exhibited plaques with significantly larger extent in the proximal, medial, and distal portion of the coronary vessels, respectively. The shortest and longest distance from the ostium of CAC is reported in Table III. Old patients exhibited higher shortest distance (Δ = +37%, p < 0.001), whereas smoking patients demonstrated a significantly higher longest distance (Δ = +5%, p = 0.038). These findings suggest a tendency for plaques to form in deeper regions of the coronary vessels among the examined groups.

**TABLE I.** Volume of the calcific plaque in the proximal, medial, and distal portion of coronary arteries. PPV = Percentage of (total) plaque volume, with respect to vessel volume. Statistically relevant *p-*values (i.e., <0.05) are highlighted in bold font.

Condition	Proximal	Medial	Distal	Total VS	PPV (%)
Volume score (mm^3^)					
Age (≥60) (*n* = 197)	19.20 [0.0; 108.1]	10.62 [0.0; 175.7]	1.33 [0.0; 57.0]	62.3 [0.0; 557.9]	1.81 [0.0; 14.2]
Age (<60) (*n* = 84)	11.86 [0.0; 101.6]	4.77 [0.0; 178.63]	0.38 [0.0; 7.98]	34.0 [0.0; 362.6]	1.16 [0.0; 12.8]
*p*-value	**0.0124**	**0.0238**	0.1871	**0.0082**	**0.0207**
Obesity (*n* = 142)	18.55 [0.0; 108.1]	10.99 [0.0; 175.7]	1.56 [0.0; 57.0]	62.2 [0.0; 557.9]	1.74 [0.0; 14.2]
No obesity (*n* = 139)	14.73 [0.0; 101.6]	7.26 [0.0; 178.63]	0.45 [0.0; 8.41]	44.9 [0.0; 403.0]	1.50 [0.0; 12.8]
*p*-value	0.2592	0.2580	0.4169	0.2958	0.5917
Smoking (*n* = 124)	17.49 [0.0; 108.1]	10.23 [0.0; 175.7]	1.20 [0.0; 27.5]	57.9 [0.0; 557.9]	1.71 [0.0; 14.2]
Nonsmoking (*n* = 157)	16.65 [0.0; 101.6]	7.87 [0.0; 178.63]	0.93 [0.0; 57.0]	50.9 [0.0; 462.2]	1.55 [0.0; 12.8]
*p*-value	0.8140	**0.0205**	**0.0268**	0.3305	0.3918
Hypertension (*n* = 183)	18.60 [0.0; 108.1]	9.60 [0.0; 175.71]	1.40 [0.0; 57.0]	59.2 [0.0; 557.9]	1.74 [0.0; 14.2]
No hypertension (*n* = 92)	13.86 [0.0; 105.9]	7.43 [0.0; 178.63]	0.36 [0.0; 9.82]	43.3 [0.0; 362.6]	1.39 [0.0; 12.8]
*p*-value	**0.0151**	0.1188	0.1642	**0.0154**	**0.0488**
Diabetes (*n* = 61)	20.62 [0.0; 86.36]	17.06 [0.0; 175.7]	2.09 [0.0; 27.53]	79.5 [0.0; 557.9]	2.26 [0.0; 14.2]
No diabetes (*n* = 220)	16.12 [0.0; 108.1]	6.87 [0.0; 178.63]	0.79 [0.0; 57.02]	47.5 [0.0; 402.9]	1.46 [0.0; 12.8]
*p*-value	**0.0223**	0.2525	0.3250	**0.0151**	**0.0188**

**TABLE II.** Extent of the calcific plaque in the proximal, medial, and distal portion of coronary arteries. Statistically relevant *p-*values (i.e., <0.05) are highlighted in bold font.

Condition	Proximal	Medial	Distal
Lesion extent (mm)			
Age (≥60) (*n* = 197)	3.06 [0.0; 9.03]	3.21 [0.0; 12.58]	2.24 [0.0; 8.01]
Age (<60) (*n* = 84)	2.64 [0.0; 12.71]	2.93 [0.0; 10.27]	2.10 [0.0; 7.32]
*p*-value	**0.0097**	0.3615	0.9555
Obesity (*n* = 142)	3.04 [0.0; 12.71]	3.43 [0.0; 12.58]	2.46 [0.0; 13.26]
No obesity (*n* = 139)	2.77 [0.0; 8.02]	2.87 [0.0; 10.27]	1.72 [0.0; 5.67]
*p*-value	0.5488	0.1989	0.1656
Smoking (*n* = 124)	2.93 [0.0; 8.71]	3.38 [0.0; 10.77]	2.43 [0.0; 7.87]
Nonsmoking (*n* = 157)	2.93 [0.0; 12.71]	2.94 [0.0; 12.58]	2.03 [0.0; 13.24]
*p*-value	0.9405	**0.0492**	0.1051
Hypertension (*n* = 183)	3.09 [0.0; 12.71]	3.23 [0.0; 12.58]	2.47 [0.0; 13.24]
No hypertension (*n* = 92)	2.63 [0.0; 8.02]	2.92 [0.0; 10.43]	1.62 [0.0; 9.01]
*p*-value	0.1110	0.3796	**0.0077**
Diabetes (*n* = 61)	3.16 [0.0; 8.27]	3.45 [0.0; 12.58]	2.48 [0.0; 7.87]
No diabetes (*n* = 220)	2.87 [0.0; 12.71]	3.07 [0.0; 10.99]	2.11 [0.0; 13.24]
*p*-value	0.2172	0.6379	0.5128

**TABLE III.** Nearest and furthest location, measured from the ostium, where coronary artery calcium formed in patients. Statistically relevant *p-*values (i.e., <0.05) are highlighted in bold font.

Condition	Shortest	Longest
Distance from ostium (mm)		
Age (≥60) (*n* = 197)	17.5 [0.0; 41.1]	67.0 [31.7; 127]
Age (<60) (*n* = 84)	12.7 [0.0; 35.2]	66.5 [33.8; 135]
*p*-value	<**0.001**	0.9583
Obesity (*n* = 142)	16.1 [0.0; 39.6]	45.9 [25.2; 72.9]
No obesity (*n* = 139)	16.3 [0.0; 41.1]	48.6 [29.6; 77.2]
*p*-value	0.8206	0.2957
Smoking (*n* = 124)	16.8 [0.0; 39.6]	68.6 [33.7; 127]
Nonsmoking (*n* = 157)	15.5 [0.0; 41.1]	65.3 [31.7; 135]
*p*-value	0.3064	**0.0383**
Hypertension (*n* = 183)	16.6 [0.0; 36.9]	68.5 [31.7; 135]
No hypertension (*n* = 92)	14.8 [0.0; 41.1]	62.7 [33.8; 114]
*p*-value	0.1175	0.4192
Diabetes (*n* = 61)	17.5 [0.0; 36.9]	72.5 [31.7; 120]
No diabetes (*n* = 220)	15.6 [0.0; 41.1]	64.9 [33.8; 135]
*p*-value	0.1678	0.2075

### D. Relation between CorT and coronary artery disease risk factors

CorT relation with CAD risk factors was then investigated. The CorT of the proximal tract resulted significantly higher both in hypertensive (p = 0.035) and obese (p = 0.024) patients (Table IV). The average tortuosity of the medial and distal tract was significantly higher in nonsmoking subjects (p < 0.05). The maximum local tortuosity and angle of tortuosity, defined as their 95th percentile to mitigate the impact of outliers, did not show any relevant difference between patients with or without any of the clinical condition considered. The incidence of patients with TS ≥ 1 (i.e., exhibiting CorT) was significantly lower (odd ratio = 0.53, *p* = 0.012) in smoking patients, while no relevant difference was assessed for the other conditions examined (Table V). Despite being not statistically relevant, a higher incidence of CorT was observed in patients with hypertension (odd ratio =1.59, *p* = 0.323) and patient with BMI < 30 (odd ratio = 0.5, *p* = 0.169).

**TABLE IV.** Tortuosity of the proximal, medial, and distal tract of the coronary arteries, between patients presenting and non-presenting a specific condition. Statistically relevant *p-*values (i.e., <0.05) are highlighted in bold font.

Condition	Proximal	Medial	Distal
Tortuosity (-)			
Age (≥60) (*n* = 197)	1.24 [1.09; 1.69]	1.18 [1.06; 1.58]	1.20 [1.05; 1.79]
Age (<60) (*n* = 84)	1.23 [1.09; 1.43]	1.17 [1.07; 1.59]	1.18 [1.05; 1.66]
*p*-value	0.4159	0.3827	0.2612
Obesity (*n* = 142)	1.26 [1.09; 1.70]	1.16 [1.07; 1.58]	1.18 [1.05; 1.67]
No obesity (*n* = 139)	1.21 [1.09; 1.79]	1.19 [1.07; 1.48]	1.19 [1.07; 1.78]
*p*-value	**0.0243**	0.6659	0.1519
Smoking (*n* = 124)	1.23 [1.09; 1.69]	1.17 [1.07; 1.58]	1.18 [1.05; 1.66]
Nonsmoking (*n* = 157)	1.25 [1.10; 1.53]	1.19 [1.06; 1.59]	1.20 [1.07; 1.79]
*p*-value	0.8097	**0.0148**	**0.0076**
Hypertension (*n* = 183)	1.25 [1.09; 1.69]	1.19 [1.06; 1.60]	1.19 [1.05; 1.79]
No hypertension (*n* = 92)	1.21 [1.10; 1.54]	1.17 [1.08; 1.58]	1.18 [1.05; 1.75]
*p*-value	**0.0353**	0.1065	0.7113
Diabetes (*n* = 61)	1.23 [1.12; 1.48]	1.20 [1.07; 1.60]	1.16 [1.07; 1.79]
No diabetes (*n* = 220)	1.24 [1.09; 1.69]	1.18 [1.07; 1.60]	1.19 [1.05; 1.75]
*p*-value	0.8038	0.5221	0.3864

**TABLE V.** Tortuosity incidence. *n* indicates the number of patients with each comorbidity that exhibits CorT (meaning a TS ≥ 1), or not. OR = odd ratio resulted from Pearson's χ^2^ test. Statistically relevant *p-*values (i.e., <0.05) are highlighted in bold font.

Condition	CorT (*n*)	Non-CorT (*n*)	OR	*p*-value
Age (≥60)	114 (69%)	76 (71%)	0.88	0.685
Obesity	78 (52%)	62 (61%)	0.58	0.169
Smoking	60 (36%)	55 (52%)	0.53	**0.012**
Hypertension	112 (68%)	68 (59%)	1.59	0.323
Diabetes	32 (20%)	22 (21%)	0.93	0.876

### E. Calcium and tortuosity features along vessel centerline

**FIG. 4.**
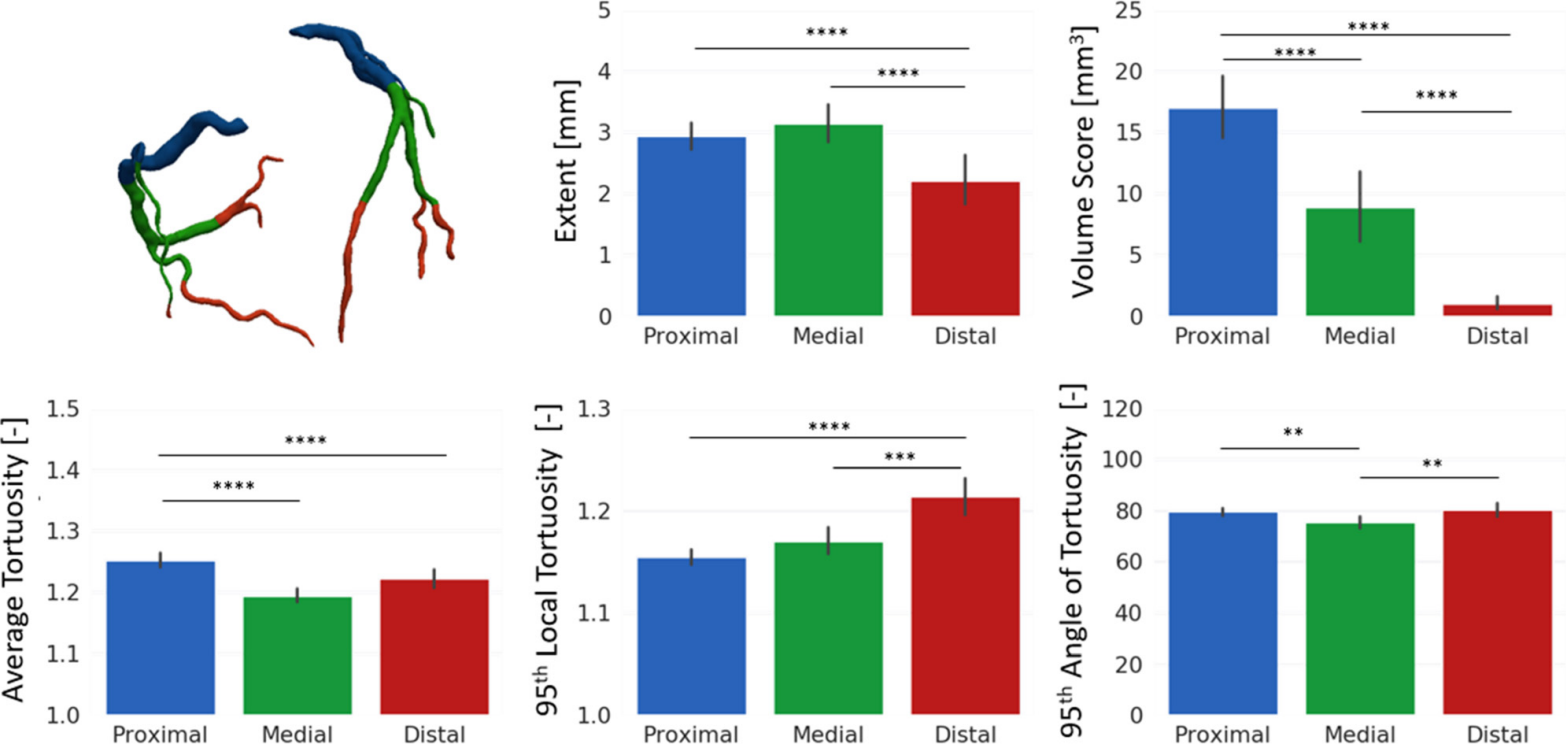
Bar plots showing calcium and vessel features in different zones of the coronary arteries. (i) First row (from left to right): model showing the three anatomical portions in exam; extent of the calcific lesion; and volume of calcific plaque. (ii) Second row (from left to right): average tortuosity of the tract; 95th percentile of local tortuosity, and 95th percentile of the angle of tortuosity. The “^*^” denotes the *p*-value of the U-test performed to compare each couple of data. ^*^ = 0.05, ^**^ = 0.01, ^***^ = 0.001, ^****^ < 0.001.

To explore the relationship between the positioning along the vessel centerline of CAC and tortuous regions an ANOVA was achieved comparing proximal, medial, and distal segments. The results of the one-way ANOVA performed on the parameters computed for calcific plaque and vessel tortuosity are summarized in Table VI and Fig. 4. All the considered features showed a significant difference (*p* < 0.001) among the anatomical portions defined. Calcium extent was significantly (*p* < 0.001) lower in the distal region, while calcium volume presented a significant (*p* < 0.001) decreasing trend, moving from proximal to distal region. The shape index of plaques showed a decreasing (*p* < 0.001) trend moving toward distal region, indicating that irregular plaques tend to form in the proximal coronary arteries. The average tortuosity of the proximal tract was found to be significantly higher (*p* < 0.001) with respect to the medial and distal tract, while the maximum local tortuosity increased significantly (*p* < 0.01) from proximal to distal region. The maximum tortuosity angle showed a significant (*p* < 0.01) decrease in the medial tract.

**TABLE VI.** Results of one-way ANOVA of the features computed in proximal, medial, and distal coronary arteries. Parameters values are reported as mean and [min; max]. Statistically relevant *p-*values (i.e., <0.05) are highlighted in bold font.

Parameter	Proximal	Medial	Distal	*p*-value
Calcium features				
Lesion extent (mm)	2.93 [0.0; 12.71]	3.14 [0.0; 12.58]	2.20 [0.0; 13.24]	<**0.001**
Volume score (mm^3^)	17.0 [0.0; 108.1]	8.87 [0.0; 178.6]	1.05 [0.0; 57.02]	<**0.001**
Tortuosity features				
Tract tortuosity	1.25 [1.09; 1.69]	1.19 [1.07; 1.60]	1.22 [1.05; 1.79]	<**0.001**
Local tortuosity (95th)	1.16 [1.03; 1.55]	1.17 [1.03; 1.61]	1.21 [1.03; 1.97]	<**0.001**
Angle of tortuosity (95th) (°)	79.6 [47.2; 118.3]	75.4 [38.4; 126.2]	80.2 [42.4; 136.6]	**0.0037**

### F. Relationship between coronary calcium and tortuosity

To determine whether any correlation subsist between coronary calcium and tortuosity, the dataset was split based on four TS thresholds (1–4) corresponding to progressively more severe coronary tortuosity (mild to severe tortuosity). For each couple of groups, the results of the statistical comparison between the features of calcium plaques are summarized in Table VII. The extent of the lesion was significantly lower in patients with a TS at least >2 (p = 0.014–p = 0.007), while the volume of the plaque was significantly lower in subject with any grading of coronary tortuosity (p = 0.004–p < 0.001). Indicating with Δ = calcium feature in patients with TS < threshold—calcium feature in patients with *TS* ≥ threshold, respectively, for each threshold value, we observed that both Δ_ext_ and Δ_vol_ increased as a higher TS threshold value was considered.

**TABLE VII.** Comparison of calcium features in patient with different grading of coronary tortuosity. For each subgroup, the number of patients with a tortuosity score (TS) below and above the threshold is reported as *n*=absolute value (percentage of the dataset). Calcium extent is expressed in mm, and calcium volume in mm^3^. Statistically relevant *p-*values (i.e., <0.05) are highlighted in bold font.

	TS < threshold	TS ≥ threshold	*p*-value
Threshold = 1	*n* = 106 (39.1 %)	*n* = 165 (60.9 %)	
Calcium extent	3.04 [0.0; 8.74]	3.03 [0.0; 13.2]	0.9701
Calcium volume	10.8 [0.0; 155.2]	7.81 [0.0; 105.9]	**0.0369**
Threshold = 2	*n* = 163 (60.1%)	*n* = 108 (39.9 %)	
Calcium extent	3.34 [0.0; 13.2]	2.42 [0.0; 12.6]	**0.0138**
Calcium volume	10.9 [0.0; 178.6]	6.02 [0.0; 175.7]	<**0.001**
Threshold = 3	*n* = 209 (77.1%)	*n* = 62 (22.9 %)	
Calcium extent	3.23 [0.0; 13.2]	1.91 [0.0; 12.6]	**0.0117**
Calcium volume	10.5 [0.0; 178.6]	3.99 [0.0; 75.3]	<**0.001**
Threshold = 4	*n* = 239 (88.2%)	*n* = 32 (11.8%)	
Calcium extent	3.12 [0.0; 13.2]	1.65 [0.0; 12.6]	**0.007**
Calcium volume	9.82 [0.0; 178.6]	2.65 [0.0; 24.8]	<**0.001**

## III. DISCUSSION

In this study, we presented a fully automated pipeline for the segmentation of coronary arteries from 3D CCTA and their characterization in terms of tortuosity and coronary calcium. We applied our pipeline to a cohort of 281 patients who underwent CCTA due to symptoms associated with CAD progression and achieved an analysis of CAC and CorT features. In general, the key innovative aspects of our study lays in (i) the robustness of the method for the automatic segmentation of coronary arteries, exploiting a large cohort of both healthy and diseased cases; (ii) the achievement of an objective method for the quantification of CorT from CCTA, accounting for vessel course three-dimensionality; (iii) the CorT and CAC characterization in different anatomical portion of the arteries and their the correlation; and (iv) the CorT and CAC characterization in different patient profiles.

Our approch exploits state-of-the-art deep learning techniques to perform coronary segmentation. The adopted CNN architecture provides end-to-end segmentation of the vessels without requiring any additional input other than the CCTA image and, thus, eliminating the bias introduced by the operator. For the coronary arteries, we obtained an average DSC, MSD, precision, and recall equal to 0.895, 0.470 mm, 0.935, and 0.861, respectively. The accuracy obtained with the yielded results is higher than that reported in previous works focused on coronary artery segmentation: Gharleghi *et al.*^18^ reported for their best model a DSC of 0.88, a precision of 0.95, and a recall of 0.82, while Gu *et al.*^21^ obtained a DSC of 0.862, a MSD of 0.61, and a precision of 0.84. Only Pan *et al.*^22^ reported a higher mean DSC equal to 0.969, which is—to the best of the authors knowledge—the best result described in the literature; the success of their approach can be attributed to the large dataset used for training (i.e., 474 CCTA) and to the fact that only healthy patients were included in their study; thus, lesions due to plaque depot and stenosis, which introduce additional variability in coronary morphology, were not considered and no test of their model on patients with CAD is reported in their work.

Our methods perform coronary artery segmentation, compute the vessel centerline, and automatically extract anatomical of interest that are clinically relevant in the planning of percutaneous coronary interventions, such as CAC burden and location, and vessel tortuosity.^23^ The analysis of such parameters generally requires specific commercial software and is performed by expert radiologists, potentially introducing inter-operator variability in the measures. Our framework does not require any input from the operator and is thus objective and repeatable in all its steps. Furthermore, by leveraging deep-learning techniques, our approach significantly accelerates the quantification of these parameters compared to the current standard evaluation routine achieved by expert radiologists, using semi-automatic tools. Indeed, our pipeline excels in efficiency, completing the automatic segmentation and quantification of CAC and CorT within about 5 min. In contrast, a comprehensive analysis conducted by an expert may demand approximately 1 h for completion, making out solution particularly suited for large population studies. The excellent results obtained in terms of DSC, MSD, precision, and recall indicates that such approach could guarantee robust and objective assessment of geometric features of the coronary arteries that are of paramount importance for clinical evaluation such as relative the position of calcification and stenosis along the vessel and tortuous regions.

The quantification of CorT features was based on a fully automated algorithm that exploits coronary centerlines information. To the best of the authors knowledge, this is the first study to describe an approach for CorT quantification from CCTA, accounting for the three-dimensionality of the vessels. In clinical practice, CorT is generally measured from invasive coronary angiography (i.e., a 2D image), introducing an error that, in general, leads to an underestimation of CorT.^24^ By leveraging the accuracy guaranteed by the deep learning-based segmentation, our approach excels in extracting the vessel centerline and identifying points that exhibit a sharp change in direction. Given that CorT is solely contingent on the vessel's morphology, ensuring accurate segmentation is paramount, and our deep learning approach excels in this task. Identifying CorT is beneficial for percutaneous intervention planning as moderate and severe CorT are known to be the cause of unsuccessful intervention and recognition of marked tortuosity before the procedure can avoid coronary vascular complications.^23^ We found a significantly higher tortuosity of the proximal tracts in hypertensive, while medial and distal tracts tortuosity was lower in smoking patients. Considering the overall TS, we observed lower incidence of CorT in smoking and obese patients, and higher incidence in hypertensive (Table V), despite the difference was not statistically relevant for obese and hypertensive subjects. Our findings agree with what is reported in different studies,^12–15^ which suggest a positive correlation between hypertension and CorT and found a negative correlation of CorT in smoking and obese patients. A main drawback of the present study, when performing statistical analysis, is the relatively limited number of cases, compared to other clinical studies in the literature, which exploited cohorts of >1000 subjects.^12,15^ With respect to such datasets, for which partial information was available, ours included CCTA for which manual expert annotation and comprehensive cardiologist analysis were both available. Still, it is worth noting that our fully automated pipeline can be potentially used for population studies on many more cases, thus overcoming the reliability issue of using a limited cohort.

In our work, the quantification of CAC was based on the method described by Pavitt *et al.*^8^ and exploits an attenuation threshold to detect calcium. Our method depends on the segmentation of the coronary vessels and the aorta. Wolterink *et al.*^25^ proposed a method for CAC segmentation based on a CNN that does not require vessel segmentations. However, the training of a CNN for CAC segmentation requires accurate manual annotation of calcification and careful inter-operator variability assessment. Moreover, by using coronary artery segmentation, it is possible to extract other CAC-related indexes of clinical interest, such as the PPV and the distance of the plaque from the ostium, that cannot be estimated by using CAC segmentation only. In our analysis, we characterized CAC based on the volume, the extent of the lesion, and the distance from the ostium. A validation of our method was conducted through both Pearson and Bland–Altman analyses, comparing volume scores obtained using our approach from CCTA with those measured from CSCT in a subset of patients. The Pearson correlation analysis demonstrated excellent agreement (r = 0.96) between the scores, and the results from the Bland–Altman analysis were consistent with previous studies that compared calcium scoring from CSCT and CCTA.^25–28^

Calcium volume and location are associated with adverse events;^6,29,30^ furthermore, CAC represents one of the most unfavorable factors limiting the success of percutaneous intervention, both due to the volume of the calcification and its extent.^23^ Our results indicate that age, hypertension, smoking, and diabetes are associated with higher CAC volume scores and PPV. This is in line with what reported in different works.^31–34^ Interestingly, the anatomical portion of the coronary arteries in which we observed significant increments of CAC was different for each factor, suggesting that specific conditions may be associated with increase in calcification in specific regions. Such results provide a new insight to further investigate in future studies. To the best of our knowledge, this is the first work to study CAC relation with different patient conditions, accounting for the position of calcium along the vessel.

The presented analysis of CAC and CorT confirmed the relationship between these markers and related comorbidities^12–15,31–34^ and a possible negative correlation between the two, previously suggested by other authors.^12,14^ We further provided a characterization of CAC and CorT based on their location along the vessel with respect to coronary ostium. Comparing CorT and CAC features in the proximal, medial, and distal coronary arteries, we found that different portions exhibit significantly different features. CAC volume and extent exhibit a decreasing pattern moving toward distal regions, while CorT tends to increase. These trends, which are coherent with what is commonly observed in medical images, suggest a negative correlation between CorT and CAC, as reported by Li *et al.*^12,14^ We further investigated the relationship between CorT and CAC by splitting our cohort based on four different TS thresholds. CAC volume and extent were significantly lower for higher values of TS, and the relative difference increased between groups as higher values of threshold were used, confirming previous results and a possible negative correlation between CAC and CorT. Our findings are not in line with those of Li *et al.*,^15^ who found no correlation between Agatston score and CorT, and Thelawi *et al.*,^13^ who reported a positive correlation. However, Thelawi *et al.* used limited dataset consisting of 83 patients, while *Li *et al.** compared CorT with a different CAC score. This represents a novelty of the present work, as no tool for such type of analysis has been described in previous studies. Since it is known that the location of adverse geometric features (e.g., tortuosity, stenosis induced by calcifications) may impact of the outcome of the therapy,^23^ our solution has the potential to further elucidate the role of coronary geometric features on patient outcome.

## IV. LIMITATIONS

In this study, we employed a deep learning-based automated pipeline to explore the correlation between coronary artery anatomical features and cardiovascular risk indicators. Limited availability of family history data for CAD, a recognized risk factor, restricted our ability to investigate correlations with the extracted parameters. All CCTA scans used for training were collected from the same center, acquired on CAD patients with the same machine, posing a limitation: training on images from diverse sources, ideally including scans from healthy volunteers, would enhance the model's generalizability. Despite this limitation, our two-stage cascaded model demonstrated successful training and promising results on our dataset, serving as a proof-of-concept for the proposed architecture's feasibility and generalization capability. While our dataset is relatively large for deep learning in coronary artery segmentation, it remains smaller than those used in population studies on CAC and CorT (exceeding 1000 patients). Because the test set was small, CorT and CAC analysis relied on manual annotation to ensure statistical significance. Nevertheless, the favorable outcomes in terms of DSC, MSD, precision, and recall suggest that applying the complete pipeline to a larger test set would yield comparable conclusions, given that CoT and CAC feature extraction is contingent solely on vessel geometry and image intensity. Although limited in size compared to such studies, our method offers potential for cohort expansion and analysis, moving toward more comprehensive patient numbers.

## V. CONCLUSION

In the present work, we have developed a novel tool for performing an objective and quantitative analysis of coronary artery anatomical features, such as tortuosity and calcifications, relevant in the definition of the best therapy for a patient and for the planning of interventions. Our cascaded CNN performed an accurate segmentation of the coronary arteries of all the test set patients, successfully segmenting stenotic regions. Our systematic analysis of CAC and CorT confirmed some relations with clinical conditions that are reported in the literature. Of note our method also provide an analysis of CAC and CorT, accounting for their location along the vessel course, thus providing a further insight in the characterization of coronary arteries anatomy that revealed specific trends for CAC and CorT features. Our tool is fully automated, quick, and reliable, and can be an innovative solution to provide clinicians with useful information to aid the decision-making process in the assessment of coronary arteries.

## VI. METHODS

### A. Dataset

In this study, 281 3D CCTA scans were retrospectively collected from Centro Cardiologico Monzino (Milano, Italy). All the subjects included underwent coronary CT due to chest pain, electrocardiogram (ECG) abnormalities, or previous cardiovascular events. The baseline characteristics of the patient cohort are summarized in Table VIII, including pre-existing comorbidities associated with cardiovascular risk and stenosis severity. The population consists of 68 females (24%) and 213 males (76%), reflecting a realistic ratio of male to female prevalence of CAD [ref.]. Images were acquired with a GE Revolution CT machine (GE Healthcare, Milwaukee, Wisconsin) with 100 kVp tube voltage and 475 mAs tube current, with ECG-triggering and contrast enhancement. For all the acquired images, dimension was 512 × 512 × 256; pixel spacing ranged from 0.365 × 0.365 to 0.4 × 0.4 mm^2^, and slice thickness ranged from 0.4 to 0.65 mm. The present study was performed in accordance with recommendations of the local Ethics Committee (R1771/22 CCM-1890), with written informed consent from all subjects, in accordance with the Declaration of Helsinki.

**TABLE VIII.** Study population baseline characteristics and coronary artery disease risk factors.

Number of patients (n)	281
Male/female (n)	213/68 (76%/24%)
Age (years)	65.3 ± 9.6
Height (cm)	169.2 ± 9.6
Weight (kg)	77.1 ± 14.5
BMI (kg/m^2^)	26.3 ± 3.0
Mean aortic pressure (mmHg)	104.4 ± 9.7
CAD risk factors	
Smoking (n)	124 (44%)
Hypertension (n)	183 (65%)
Diabetic (n)	61 (22%)
Hypercholesterolemia (n)	188 (67%)
LDL (mmol/l)	4.18 ± 0.40
HDL (mmol/l)	1.36 ± 0.93
Stenosis grading	
Mild (n)	118 (42%)
Moderate (n)	205 (73%)
Severe (n)	63 (23%)

#### 1. Manual annotation

**FIG. 5.**
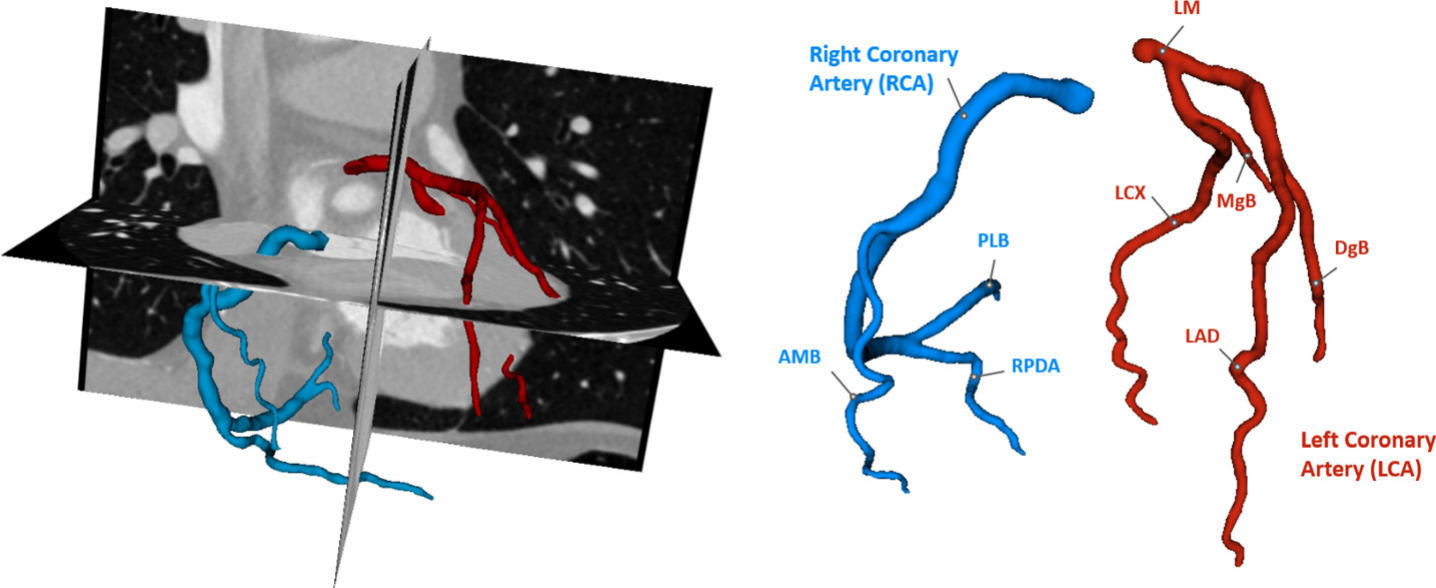
Example of manual annotation on CCTA, reporting the branches included the segmentations. AMB = acute marginal branch; DgB = diagonal branch; LAD = anterior descending artery; LCX = circumflex artery; LM = left main trunk; MgB = marginal branch; PLB = posterolateral branch; and RPDA = right posterior descending artery.

Each CCTA scans was manually annotated using 3D Slicer^35^ by two bioengineering experts, possessing 4 and 7 years of experience in medical imaging analysis, respectively, under cardiology experts' supervision. The segmentation was performed using a gold-standard segmentation method, by applying a thresholding filter with a threshold value chosen by the operator, to isolate the coronary arteries lumen, followed by manual correction of the contour of the vessels where over- and under-segmented.^18^ Each annotation included the main coronary branches reported in Fig. 5, other branches were considered only whether their caliber was >1.5 mm. Segmentation was interrupted when the caliber of the vessel was <1.5 mm, as branches smaller than 1.5 mm are not clinically relevant.^36^

### B. Neural network training

**FIG. 6.**
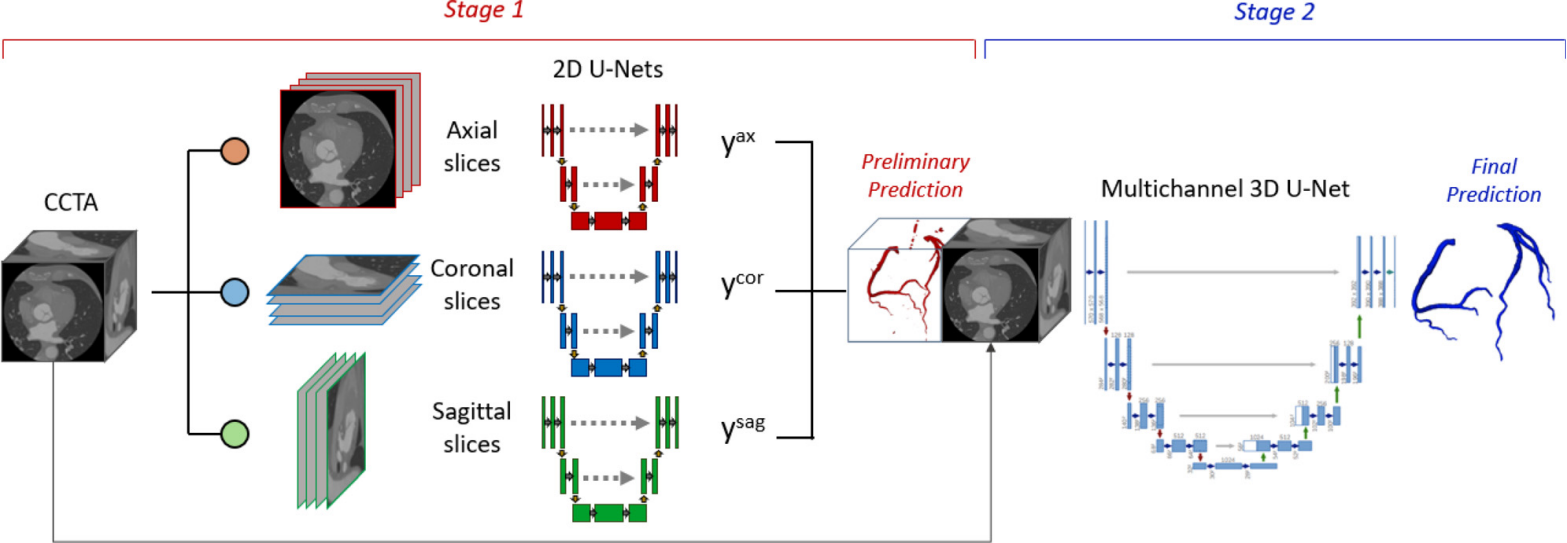
General framework of our cascaded CNN. y^ax^, y^cor^, and y^sag^ denote the posterior probabilities produced by the models trained on axial, coronal, and sagittal images, respectively. In stage 1, three 2D U-Nets receive as input the 2D CCTA slices from different directions. The combined prediction is concatenated to the original CCTA and used to train, in stage 2, a multichannel 3D U-Net.

The dataset was split into a training and a test sets: 238 (85%) randomly chosen cases were used to train the CNN, while the 43 remaining cases (15%) were used for testing (demographics are available in the supplementary material). In the present work, we adopted a two-stage cascaded approach (Fig. 6), based on the nnU-Net framework, that combines a multi-view 2.5D network (i.e., a network trained on 2D images used to reconstruct 3D volumes by exploiting the spatial positioning of the 2D slices),^37^ capable of managing images with large dimensions, and a 3D network that accounts for the connectivity between the slices. In the first stage, each CCTA image was sliced along the three orthogonal acquisition directions, namely, the sagittal, coronal, and axial directions, resulting in three independent 2D datasets. Three models were trained, then the resulting posterior probabilities tensors were combined and converted to a binary segmentation. In the second stage, a 3D model was trained using as input the original CCTA image concatenated to the segmentation obtained in first stage. The model was implemented in Pytorch, using MONAI framework utilities.

#### 1. Stage 1 (S1)

In the first stage, a multi-view 2.5D model was implemented. Three 2D models were trained using images sliced along orthogonal directions (i.e., sagittal, coronal, and axial directions). The trained models were based on the 2D-Unet architecture: the downsampling path consisted of five 3 × 3 convolutional layers, consisting in a convolution, an instance normalization, and PreLU activation; the upsampling path consisted of five upsampling layer concatenated to the corresponding feature map of the downsampling path and followed by a convolutional layer. To increase the variability of each of the three datasets, a data augmentation routine was implemented. Image intensity was scaled in the interval [−30, 800] HU and normalized to zero mean, then Gaussian smoothing and affine transformation were applied with random probability. Due to the large class imbalance between coronary and background voxels, a weighted combination of the Dice and Focal Loss (DFL) was used to train each model.

$$\mathrm{DFL}\left(y,\hat{y}\right)=\lambda_{F}\left[-\alpha {\left(1-{\hat{y}}_{i}\right)}^{\gamma }y_{i}\;\text{log}\;{\hat{y}}_{i}-\left(1-\alpha \right){\hat{y}}_{i}^{\gamma }\left(1-y_{i}\right)\text{log}\left(1-{\hat{y}}_{i}\right)\right]+\lambda_{D}\left[1-\frac{2\sum_{i}y_{i}{\hat{y}}_{i}}{\sum_{i}y_{i}+\sum_{i}{\hat{y}}_{i}}\right],$$

where $y_{i}$ is the probability of the ground truth and ${\hat{y}}_{i}$ is the posterior probability of the i-th voxel to belong to the segmented class. To mitigate class imbalance α=0.6 and γ=2 were used, as in the work by Pan *et al.*^22^ The weights were set to $\lambda_{F}=1.1,\;\lambda_{D}=0.4$. The training of each model was performed processing groups of 32 (i.e., batch size) slices separately, for 600 training epochs, on 2 20GB NVIDIA A100 graphic processing units (GPUs). Once each model was trained, the posterior probability tensors resulting from inference on the input image were combined by taking the element-wise maximum of the foreground class and finally converted to a one-hot binary label map, producing a rough preliminary segmentation of the coronary arteries.

#### 2. Stage 2 (S2)

In the second stage, the rough segmentation obtained in S1 was concatenated to the original volumetric image and used as input to train a 3D model, based on the 3D U-Net architecture. The network architecture was analogous to the 2D models implemented in S1. Differently from such models, this network performs convolutions of the processed tensors with 3D filters. Convolutional kernel size was set to 5 × 5 × 5, and batch normalization was adopted. In addition to the transformations described in data augmentation routine for S1, image orientation was set to LPS for all the images, and random 64 × 64 × 6 image patches were cropped and passed to the model, as the entire volumetric image could not fit into GPU memory. The 3D model was trained with a batch-based approach, setting the batch size to 8. The DFL was used as loss function, with weights $\lambda_{F}=0.5,\;\lambda_{D}=1$.

### C. Performance evaluation

To assess the performance of the model, the DSC, the mean surface distance (MSD), precision and recall were evaluated using the test set data. These metrics are commonly used in segmentation problems and are defined as follows:

$$\mathrm{DSC}=\frac{2\cdot TP}{2\cdot TP+FP+FN},$$

where TP, FP, and FN are the number of true positive, false positive, and false negative, respectively. DSC measure the overlap between the segmentation and the ground truth reference,

$$\mathrm{MSD}=\frac{1}{n_{S}+n_{S^{\prime }}}\left(\sum_{i=1}^{n_{S}}d\left(p_{i},\;S^{\prime }\right)+\sum_{i=1}^{n_{S^{\prime }}}d\left(p_{i}^{\prime },\;S\right)\right),$$

where **p** and **p**′ denote the points of surface S and S′ respectively, and $d\left(p,S^{\prime }\right)=\underset{p^{\prime }\in S^{\prime }}{\text{min}}\left\|p-p\prime \right\|$. MSD evaluate, averagely, how close points on the segmentation and the ground truth are, measuring the accuracy at the boundary of the coronary arteries.

$$\mathrm{Precision}=\frac{TP}{TP+FP},$$

$$\mathrm{Recall}=\frac{TP}{TP+FN}.$$

Precision and recall quantify the rate of relevant elements (i.e., voxel) segmented, with respect to all the retrieved elements and all the relevant elements, respectively.

### D. Pipeline implementation

To achieve anatomical characterization of the coronary arteries, a fully automated pipeline was implemented, embedding the cascaded-CNN described. After the automatic segmentation of the coronary arteries from CCTA, the following steps are sequentially performed: (i) detection of vessel centerlines; (ii) analysis of coronary artery tortuosity; (iii) detection of calcific lesion; and (iv) CAC quantification.

#### 1. Extraction of vessel centerlines

**FIG. 7.**
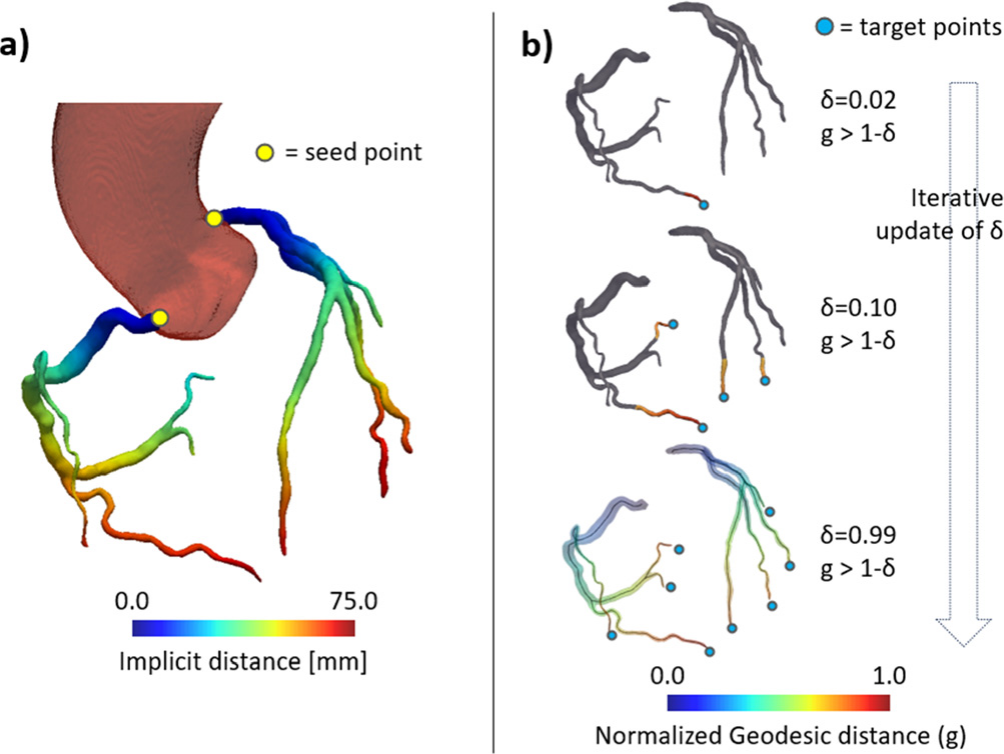
Representation of the framework adopted for the automated extraction of centerlines. (a) Maps of the implicit distance from the aorta. Seeds points (yellow dots) are defined as the point on each coronary artery that minimize implicit distance. (b) Recursive detection of target points and extraction of centerlines.

To perform a comprehensive analysis of CorT and CAC, the centerlines of the vessels were extracted. First, triangulated surface meshes were reconstructed from the binary segmentation, using a marching cubes algorithm. Centerlines were computed using the Vascular Modeling Toolkit (VMTK) library. To extract the centerline of a vessel, seeds and target points must be provided. For each coronary artery tree, the seed point corresponding to the ostium was identified as the closest point to the aorta [Fig. 7(a)], segmented automatically by our previously trained model.^38^ Target points were identified using a recursive thresholding procedure similar to the one described by Saitta *et al.*^39^ Briefly, for each point in the vessel tree surface, the geodesic distance to the ostium ( g) was computed and normalized between 0 and 1. Then, the surface points where g is higher than 1-δ are selected through thresholding. For each connected region in the thresholding output, the point with highest g is selected as a centerline end point. By gradually increasing δ, more regions fall below the selected threshold until all unique vessel tree end points are identified [Fig. 7(b)]. Once the centerline extraction was completed, based on the value of g, three anatomical portions of the coronary arteries were defined: the proximal zone ( g ≤ 0.33), the medial zone (0.33 < g ≤ 0.66), and the distal zone ( g > 0.66).

#### 2. Analysis of coronary artery tortuosity

**FIG. 8.**
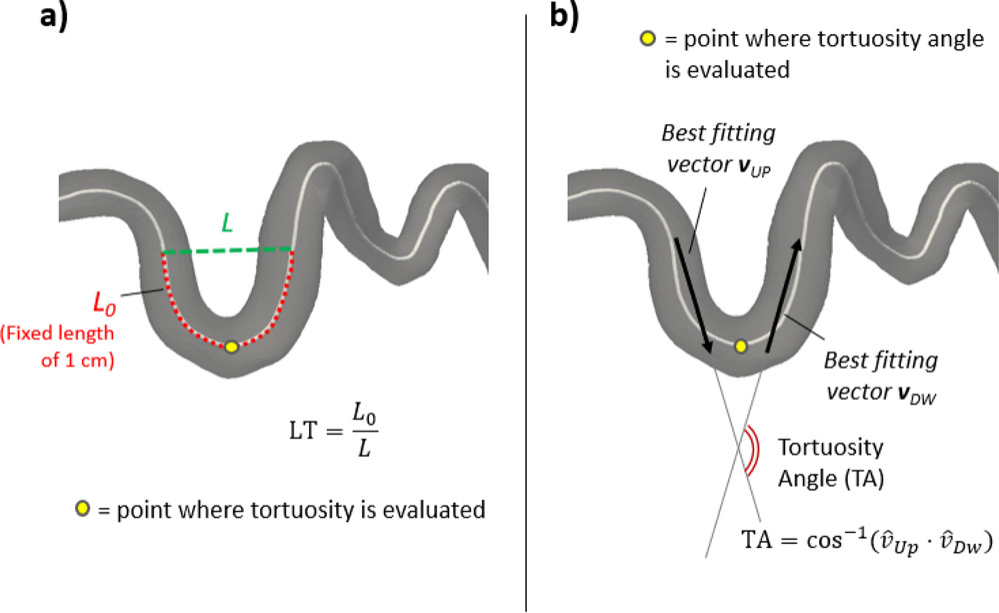
Coronary tortuosity analysis workflow. (a) Pointwise evaluation of local tortuosity, defined as arc length (L_0_)/minimum distance (L) ratio, using a reference arc of length 1 cm. (b) Pointwise evaluation of the tortuosity angle, based on the best fitting vectors of the halves of the reference arc upstream and downstream the point.

After centerline extraction, an analysis of the CorT was achieved. Tortuosity was defined as the ratio of actual path length ( $L_{0}$) to the straight distance ( L) between the ends of the path. The local tortuosity (LT) was defined pointwise along the centerlines, considering reference arcs of length 1 cm, centered in the point where tortuosity was being computed [Fig. 8(a)]. Tortuosity angle (TA) was also evaluated pointwise by computing the arccosine of the dot product of the vectors defined by the straight lines that fitted the upstream ( ${\hat{v}}_{Up}$) and downstream ( ${\hat{v}}_{Dw}$) arc [Fig. 8(b)] in a least-square sense

$$TA={\text{cos}}^{-1}\left({\hat{v}}_{Up}\cdot {\hat{v}}_{Dw}\right).$$

Finally, the tortuosity score was computed for each subject. TS was defined as the number of branches that presented at least three bends along their course with TA ≥ 45°.

#### 3. Analysis of calcific lesions

To automatically detect calcific lesions from CCTA, a patient-specific attenuation threshold $\theta_{HU}$ for calcium was determined as $\theta_{HU}=\mu \left(Ao_{HU}\right)+2.5\sigma \left(Ao_{HU}\right),$^8,25^ where $\mu \left(Ao_{HU}\right)$ and $\sigma \left(Ao_{HU}\right)$ indicate the mean and the standard deviation of the attenuation range in the aorta, respectively. CAC was identified by thresholding the original image to $\theta_{HU}$, within the coronary artery volume. Then, a region growing algorithm was applied to each detected lesion, making the calcium plaques expand in the neighboring pixels, if their attenuation is above the 98th percentile of the attenuation range in the coronary arteries. To exclude image artifacts, lesions of dimension below 1 mm^2^ were removed.^40,41^

CAC was characterized in terms of volume, extent of the plaque and distance from the coronary ostium. To compute the position of each lesion along the vessel, CAC was projected onto the coronary centerlines. The distance of each lesion from the coronary ostium was defined as the minimum abscissa value of the centerline on which the plaque was projected. The extent of the lesion was defined as the difference between the maximum and minimum abscissa value.

### E. Statistical analysis

Statistical analyses were performed using the Python SciPy 1.9.1 statistics library. The Shapiro-Wilk test was performed to assess data normality. Normally distributed data are reported as mean ± standard deviation; non-normally distributed data are reported as median [min; max]. CorT and CAC features were compared in subgroups of patients, defined based on the existence of the cardiovascular risk factors listed in Table VIII. Specifically, the cohort was split based on old age (i.e., >60), obesity (i.e., body mass index > 30), smoking habit, hypertension, and diabetes (any type). Comparison of continuous variables between each couple of groups was performed using the Mann–Whitney *U* test. Categorical variables (i.e., the TS) were compared using Pearson's χ^2^ test. CorT and CAC features were then compared among the proximal, medial, and distal portion of the coronary arteries, by performing a one-way ANOVA. Finally, CAC features were compared in relation to CorT: the dataset was split into subgroups based on a TS threshold that varied between 1 (mild tortuosity) and 4 (severe tortuosity) and, for each subgroup, the comparison between calcium features in each subgroup was achieved with Mann–Whitney *U* test. For all the tests, a *p* value below 0.05 was considered statistically significant.

#### 1. Validation of calcific lesion analysis

To validate the implemented CAC analysis, a subset of 13 patients with both CSCT and CCTA data available was selected. Calcific lesions were manually annotated by an expert on CSCT by identifying connected pixels with intensity above 130 HU^7^ and the VS was computed. The agreement between the two sets of scores was assessed through a Pearson correlation analysis and a Bland–Altman analysis.

## SUPPLEMENTARY MATERIAL

See the supplementary material for details of (i) training and test set baseline demographic characteristics, (ii) the CLAIM form^42^ filled in, and (iii) the GDPR protocol for the approval of this study.

## Data Availability

The data that support the findings of this study are available from the corresponding author upon reasonable request.
